# Systematic review on the effects of exercise with and without breakfast consumption on cognitive performance in healthy adults

**DOI:** 10.1186/s40359-024-02327-y

**Published:** 2025-01-10

**Authors:** Shu-Shih Hsieh, Yu Tian, Chun-Yuan Cheng, Yung-Chih Chen

**Affiliations:** 1 Department of Psychology, Kingston University London, Kingston upon Thames, UK; 2 Department of Physical Education and Sport Sciences, National Taiwan Normal University, No. 162, Sec. 1, Heping E. Rd, Taipei, Taiwan

**Keywords:** Breakfast, Overnight fasting, Exercise, Processing speed, Executive function

## Abstract

**Background:**

The objective of this systematic review was to review the current evidence on the effects of acute exercise with and without morning breakfast consumption on cognitive performance.

**Methods:**

This systematic review followed the Preferred Reporting Items for Systematic Reviews and Meta-Analysis guidelines and is registered in the International Prospective Register of Systematic Reviews (CRD42023396125). Studies were included if they investigated effects of acute exercise with and without preceding morning breakfast on cognitive performance measured during and following exercise in healthy adults. Eligible studies from 5 electronic databases, PubMed, Scopus, MEDLINE, Web of Science, and Embase, with no limitations on years and dates of publications to retrieve maximal number of literature (literature search and screen were completed on 13 December 2024). Study quality was assessed using the Physiotherapy Evidence Database scale (PEDro).

**Results:**

A total of 3018 studies were screened. Five studies, involving 70 participants (42 women, aged between 18 and 50 years) in total (sample size per study: 10–24), were eligible for inclusion in this review. The synthesised results based on 5 identified studies with healthy adults showed that there was no indication that effects of exercise on cognitive performance (e.g., processing speed, inhibitory control) are altered by breakfast skipping and/or consumption (e.g., different portion, macronutrients, and contents). The included studies had a mean PEDro score of 4.0 (scored between 3 and 5), suggesting ‘fair’ methodological quality.

**Conclusion:**

The synthesised results showed that there was no indication that effects of exercise on cognitive performance (e.g., processing speed, inhibitory control) are altered by morning breakfast consumption or macronutrients and contents of breakfast in healthy adults. However, the synthesised results should be interpreted cautiously, given the limited evidence and the heterogeneity in methodology with mostly involved young and healthy adults. Further investigation regarding interactive effects of breakfast and exercise on cognition, especially in individuals with metabolic disease or medical conditions, is warranted.

**Supplementary Information:**

The online version contains supplementary material available at 10.1186/s40359-024-02327-y.

## Background

Performance in different cognitive domains, such as attention and vigilance, executive functions (EFs), and memory, is critical for optimising daily functioning, work and school productivity, and quality of life [1, 2]. In classrooms and offices, for example, individuals require attention to focus on relevant information while relying on distinct EFs to suppress irrelevant distraction (inhibitory control), hold and mentally organise information (working memory), and adjust their behaviour or thoughts based on updated demands or priorities (cognitive flexibility) to achieve goals [3–5]. These abilities have profound attribution effects on academic attainment and job performance [6, 7]. A healthy lifestyle considering exercise and breakfast have been shown effective in improving various health domains, such as better cardiometabolic [8, 9] and vascular health [10]. Of note, the beneficial effects of either exercise participation [11] or breakfast consumption [12, 13] can be extended to better cognition, including attention and vigilance, executive function, and recognition of memory.

Of note, it is well-documented that exercise results in neural adaptations and a series of neurophysiological and metabolic changes in the brain, such as increased secretion of brain derived neurotrophic factors (BDNF) and decreased oxidative stress and inflammatory factors (e.g., Tumour Necrosis Factor-Alpha [TNFα], Interleukin 6 [IL-6]) in the brain, as well as increased blood flow perfusion [14], and increased uptake of glucose [15]. Neuroimaging studies further indicated that exercise improves attention resources allocation measured by electroencephalogram (EEG) [16] and induces better connectivity between cortical regions subserving higher-order cognitions (e.g., attention, memory, decision-making) during a cognitively challenging task [17]. At a behavioural level, meta-analysis also showed a small to moderate effect of exercise on performance of processing speed/vigilance, attention, executive function, and memory across the lifespan [11]. The majority of research supports the modulatory effects of exercise at a moderate intensity [11] whereas recent studies further shed light on the beneficial effects of high-intensity exercise to executive function and attention [18].

In addition to exercise, energy intake and meal consumption may also play a vital role in cognitive performance. In particular to cognition, previous research showed that omission of breakfast led to reduced performance on attention and long-term memory, and these impairments increased in magnitude over the morning [19]. In contrast, breakfast consumption could induce various neurobiological and neurophysiological adaptations in the brain, such as increased glucose uptakes that would favour memory performance and response speed [20], as well as a more favourable state of dynamic cerebral autoregulation [21]. These breakfast-induced adaptations may, in turn, foster cognitive performance, and research has shown that such effects could last 30–60 min after the meal [13]. The modulatory effects of breakfast consumption on subsequent cognitive performance could be explained by the effects of specific macronutrients that compose the meal. For instance, carbohydrate intake may improve performance on task-measured attention inhibition and attention resources allocation measure by EEG, and increased blood glucose levels may mediate the positive relation between carbohydrate intake on attention resources allocation [22].

Based on the above outlined effects of exercise and breakfast on cognition, it is reasonable to hypothesise an interaction effect between morning breakfast and exercise on cognitive performance, such that individuals would have better cognitive performance under a fed exercise state as compared to an overnight fasting exercise state, due to a synergistic effect of combining breakfast with exercise. Several published research examined the interactive effects of acute bouts of exercise in combination with pre-exercise morning breakfast on task-measured cognitive performance [23–27]. A better understanding of this interaction and synergistic effects can possibly inform greater lifestyle choices and arrangements that could maximise cognitive performance throughout the day. However, to date there is no existing review that systematically evaluates the effects reported by individual studies and assesses the quality of published research. As such, the objective of this review was to systematically evaluate whether morning breakfast consumption and/or omission (i.e., extended overnight fasting) could alter the effects of exercise on cognitive performance. The goal of this systematic review is to serve as a reference which identified strengths and limitations of published research and guide future research investigating the effects of exercise with or without breakfast consumption on cognitive performance.

## Methods

This review was performed in accordance with the Preferred Reporting Items for Systematic Reviews and Meta-Analysis (PRISMA) guidelines [28, 29] and is registered in the PROSPERO (International Prospective Register of Systematic Reviews) with the registration number (identification code: CRD42023396125). Please see Supplement 1 for PRISMA checklist.

### Eligibility criteria

The Population, Intervention, Comparison, Outcome and Study (PICOS) framework [29] was used to determine the inclusion criteria for studies as follows: (P) Participants: participants were healthy without any cardiovascular diseases, history of brain injury, or psychiatric or neurological disorder; (I) Intervention: exercise interventions preceded by breakfast consumption (Breakfast-Ex) and with clearly defined modality (e.g., continuous or intermittent exercise), intensity (e.g., V̇O_2max_ or heart-rate), duration, and/or volume (e.g., energy expenditure). Breakfast was defined as having consumption of meals containing energy with any of macronutrients (e.g., carbohydrate, protein, and fat) before 11:00 [30] (C) Comparator: studies included fasted exercise condition (Fasted-Ex); (O) Outcome: studies tested cognitive performance by computerised or paper- and pencil-based neuropsychological assessments at baseline, before, during exercise, and/or after exercise; (S) Study design: studies were withing-subjects, randomised crossover trials.

Studies were excluded if they (1) did not include a fasted exercise condition, (2) did not explicitly state the parameters of exercise, such as type, intensity, and duration; (3) included nutrition supplements during and/or following exercise which may cause confounding effects to the during- and post-exercise cognitive responses, and (4) were not published in an English peer-reviewed journal.

### Information sources and search strategy

**Fig. 1:**
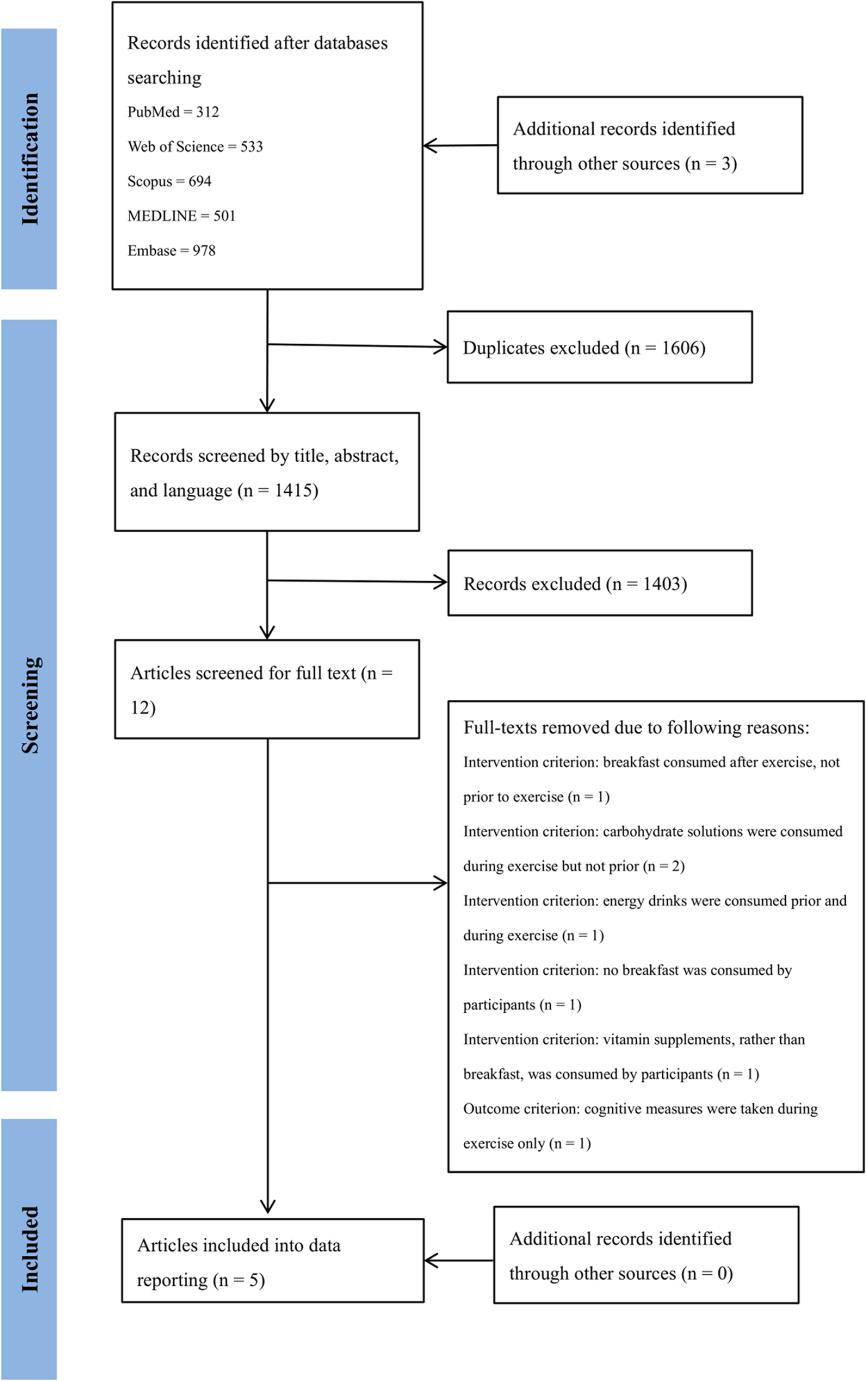
PRISMA diagram of study selection and screening

Five electronic databases, PubMed, Scopus, MEDLINE, Web of Science, and Embase were searched for eligible studies from inception. In order to retrieve the maximal number of available literatures for selection and screening, we did not limit years and dates in literature search and retrieval (all literature search and screening was completed on 13 December 2024). The keywords used to search the titles and abstracts were discussed by the research team to maximise the chance of identifying relevant articles. The following keywords were used: (“*breakfast*” OR “fast*” OR “*carbohydrate*”) AND (“*cognition*” OR “*cognitive function*” OR “*executive function*” OR “*memory*”) AND (“*exercise*” OR “Physical activit*”). The inclusion and exclusion of articles were decided according to the PICOS criteria, with the screening and selection of studies being completed by two authors independently (YT and CYC). First, the titles and abstracts were independently assessed by these two authors and initially coded as ‘yes’, ‘no’ or ‘maybe’ for inclusion. The same two authors then reviewed the full texts of the ‘yes’ and ‘maybe’ studies, and disagreements regarding the inclusion of any study were resolved by discussion with a third reviewer (YCC). The reference lists of all the included articles were then searched to check for potentially relevant studies. Figure 1 provides an overview of the selection process.

### Data collection and items

The data collection was conducted by the same two authors (YT and CYC). The authors thoroughly read the included studies and extracted the following data: (1) first author’s name, publication year, and country of data collection; (2) participants’ characteristics (sample size, age, weight status, aerobic fitness measured by V̇O_2max_); (3) study design (i.e., experimental manipulation, calorie intake during breakfast) and; (4) details of the exercise intervention, (5) outcome measures for cognition, and (6) the main research findings. Two studies [24, 27]included more than one cognitive test during post-exercise recovery, and some of the tests were preceded by either drinks or lunch that were unrelated to breakfast. In order to minimise the confounding effects from non-breakfast related energy intake to post-exercise cognitive responses, we only extracted results from cognitive measures not preceded by non-breakfast related energy intake.

### Synthesis methods

The limited number of available studies, coupled with the high heterogeneity observed among them, precluded us from conducting a meta-analysis. As such, the results presented in this review were analysed using a narrative synthesis approach. The data were first analysed by one reviewer (SSH) and then verified by a second reviewer (YCC). As all the necessary information were obtained from the articles, no authors were contacted for information. A *p* value of < 0.05, presented in the original studies, was used across the studies to determine the significant effects of exercise with and without breakfast intake on cognition. The results were summarized in Table 1.

**Table 1:** Summary of included studies

Authors	Participants	Design	Experimental manipulation	Exercise	Outcome measures	Results
Paul et al. [23] US	*N* = 12 (6 women) health adults Age: 25 ± 3 years BMI: 22 ± 2 kg/m^2^ V̇O_2max_: 54 ± 6 mL·kg^−1^·min^−1^	Randomised crossover	Trial started at 7:00 a.m. (fasting 12 h) Calorie consumed during breakfast: women 281 kcal; men 368 kcal Conditions: 1) Fasted exercise 2) Corn breakfast exercise 3) Wheat breakfast exercise 4) Oat breakfast exercise	**M**: ergometer cycling **I**: 60% V̇O_2max_ **D**: 90 min	*Baseline and within 1 h after interventions*: **Processing speed**: digit symbol substitution test, multiple-choice reaction time task **Divided attention**: divided attention task	**No significant change** in cognition between experimental trials within 1 h following intervention
Veasey et al. [27] UK	*N* = 12 active healthy men Age: 23 ± 2 years BMI: 25 ± 2 kg/m^2^ V̇O_2max_: 51 ± 1 mL·kg^−1^·min^−1^	Randomised crossover	Trial started at 7:15 − 7:45 a.m. (fasting 12 h) Calorie consumed during breakfast: 451 kcal Conditions: 1) Fasted rest 2) Fasted exercise 3) Breakfast rest 4) Breakfast exercise	**M**: treadmill running **I**: 60% V̇O_2max_ **D**: exercised until ~ 700 kcal was expended	*Baseline and immediately after interventions*: **Processing speed**: simple and choice reaction time task **Sustained attention**: rapid visual information processing task **Inhibitory control**: Stroop test **Working memory**: n-back task, rapid visual information processing task	**No significant change** in cognition between experimental trials immediately following interventions
Veasey et al. [24] UK	*N* = 24 active healthy women Age: 21 ± 2 years BMI: 22 ± 2 kg/m^2^ V̇O_2max_: not reported	Randomised crossover	Trials started at 8:00–8:30 a.m. (fasting 12 h) Calorie consumed during breakfast: low-caloric breakfast 118 kcal; high-caloric breakfast 236 kcal Conditions: 1) Fasted exercise 2) Low-caloric breakfast exercise 3) High-caloric breakfast exercise	**M**: treadmill running **I**: 65% HRR **D**: 30 min	*Baseline and 2 h after interventions*: **Processing speed**: 4-choice reaction time task **Sustained attention**: rapid visual information processing task **Inhibitory control**: Stroop test **Working memory**: n-back task, rapid visual information processing task	**No significant change** in cognition between experimental trials within 2 h following intervention
Komiyama et al. [26] Japan	*N* = 10 active healthy men Age: 22 ± 2 years BMI: 24 ± 2 kg/m^2^ V̇O_2max_: not reported	Randomised crossover	Trials started at 7:00 a.m. (fasting 12 h) Calorie consumed during breakfast: 350 kcal Conditions: 1) Fasted exercise 2) Breakfast exercise	**M**: ergometer cycling **I**: intensity corresponded to 140 beats.min^− 1^ **D**: 30 min	*Baseline and during interventions*: **Processing speed**: Go trials in Go/NoGo task **Working memory**: spatial delayed response task **Inhibitory control**: NoGo trials in Go/NoGo task	**Processing speed** (Go RT) improved by exercise regardless of breakfast consumption. **Accuracy of processing speed** (Go accuracy) improved immediately after breakfast (before exercise) in the breakfast fed condition. **Accuracy of processing speed** (Go accuracy) improved only in the fasted exercise trial. **No significant change** in inhibitory control and working memory.
Kotopoulea-Nikolaidi et al. [25] UK	*N* = 12 women (perimenopausal) Age: 46 ± 2 years BMI: 25 ± 3 kg/m^2^ V̇O_2max_: 30 ± 5 mL·kg^−1^·min^−1^	Randomised crossover	Trials started 9:00 am (fasting 11 h) Calorie consumed during breakfast: 500 kcal Conditions: 1) Fasted exercise 2) High-carbohydrate breakfast exercise 3) High-protein breakfast exercise	**M**: intermittent uphill walking/jogging, 4-min exercise + 3-min recovery per bout x 4 bouts **D**: 28 min in total **I**: 85–90% HR_max_ for exercise and 50–60% HR_max_ for recovery	*Baseline and after interventions (time lapse between exercise termination and follow-up cognitive test not specified)*: **Inhibitory control**: Stroop test **Cognitive flexibility**: shift Stroop test **Working memory**: n-back	Improved **inhibitory control** (Stroop RT) improved by exercise regardless of breakfast consumption. Improved **cognitive flexibility** (shift Stroop RT) improved by exercise regardless of breakfast consumption. **No significant change** in working memory

### Quality assessment

The quality assessment of the included studies was conducted by utilizing the Physiotherapy Evidence Database (PEDro) scale [31]. The scale assigns a score out of 10 as a quantitative assessment of bias, with higher scores indicating better methodological quality. The PEDro evaluates several types of bias, including allocation bias (randomization and concealment of allocation), performance bias (blinding of participants and personnel), and detection bias (blinding of outcome assessment). Additionally, the scale considers other factors such as eligibility criteria, baseline comparability, retention rate, intention-to-treat analysis, between- group statistical comparisons for at least one key outcome, as well as point and variability measures. If the PEDro score was not available from the database for a study, the two review authors (SSH and YT) independently calculated the score. Any discrepancies were resolved through discussion, and if needed, a third author (YCC) served as an adjudicator.

## Results

### Study selection

Figure 1 illustrates the study selection process in a PRISMA flow diagram. A total of 3018 studies were initially identified from PubMed, Scopus, MEDLINE, Web of Science, and Embase. An additional 3 studies were identified through further reading. Of these, 1066 duplicates were removed. Accordingly, a total of 1415 articles were screened for title and abstract. After title and abstract screening, 12 articles were selected for full-text screening, and resulted in a total of 5 full-text articles included in the data extraction and reporting. See Fig. 1 for PRISMA flowchart of study selection and Table 1 for summary of study characteristics.

### Study characteristics

The 5 included articles all employed within-subjects, randomised crossover design, with a total of 28 male and 42 female participants aged between 19 and 50 years old (sample size per study: 10–24). Participants had a mean body mass index (BMI) between 20 and 28 kg/m^2^, indicating that the majority of participants were non-obese. Among the included studies, 3 studies [23, 25, 27] provided information on V̇O_2max_, with mean V̇O_2max_ between 25 and 60 mL·kg^−1^·min^−1^, suggesting that the fitness levels of participants varied across studies.

In terms of research protocol, all studies commenced the trials at 7–9 am, with participants having an overnight fasting between 11 and 12 h [23–27]. The calorie consumption during breakfast among male participants was generally consistent across studies (350–451 kcal) [23, 26, 27]; there was considerable variation in calorie consumption during breakfast among female participants across studies (118–500 kcal) [23–25]. Two studies compared the effects of Fasted-Ex and Breakfast-Ex [26, 27], one study compared Fasted-Ex to Breakfast-Ex with different types of breakfast (corn, wheat, and oat intake) [23], one study compared Fasted-Ex and to low- and high-caloric Breakfast-Ex trials [24], and one study investigated the differential effects across Fasted-Ex and Breakfast-Ex with either high-carbohydrate or high protein intake [25].

With regard to the exercise intervention, 2 studies involved continuous treadmill running [24, 27], 2 studies involved continuous ergometer cycling [23, 26], and one study involved high-intensity intermittent exercise, with 4 bouts of 4-minute intermittent uphill walking/jogging interspersed by 3-minute recovery [25]. In terms of metrics to quantify exercise intensity, 2 studies used V̇O_2max_ (e.g., 60% V̇O_2max_) [23, 27], one study used % HR_max_ (e.g., 85–90% HR_max_) [25], one study used % HRR (e.g., 65% HRR) [24], and one study used a fixed heart rate criterion (e.g., 140 beats per minute) [26]. Regardless of the metrics used, the intensities of exercise were all corresponding to a moderate-to-vigorous level. Relative to duration of exercise bout, 3 studies had a duration of 28–30 min [24–26], one study had a prolonged duration of 90 min [23], and another study had participants exercise until a total of 700 kcal were spent [27].

Relative to the different subcomponents of cognition, 4 studies assessed processing speed [23, 24, 26, 27], 2 studies assessed sustained attention [24, 27], one study assessed divided attention [23], 4 studies measured inhibitory control [24–27], 4 studies measured working memory [24–27], and only one study tested cognitive flexibility [25]. While most studies measured cognitive performance at baseline and post exercise, one study measured performance on processing speed, working memory, and inhibitory control at baseline and during exercise [26]. Specifically regarding 4 studies with a pretest-posttest design [23–25, 27], our synthesised strategy to exclude cognitive measures preceded by non-breakfast related energy intakes (e.g., drinks or lunch) resulted in that we only retrieved cognitive measures up to 2 h following exercise. Table 2 summarises different subdomains of cognition as well as the specific tasks utilised to assess them by included studies.

**Table 2:** Subcomponents of cognition measured and neuropsychological tasks utilised by included studies

Subcomponent	Tasks	Reference
Basic information processing	Simple reaction time Choice reaction time Digit symbol substitution Go trials of Go/NoGo	Paul et al. [23] Veasey et al. [24, 27] Komiyama et al. [26]
Sustained attention	Rapid visual information processing	Veasey et al. [24, 27]
Divided attention	Divided attention	Paul et al. [23]
Inhibitory control	Stroop NoGo trials in Go/NoGo	Veasey et al. [24, 27] Komiyama et al. [26] Kotopoulea-Nikolaidi et al. [25]
Working memory	N-back Rapid visual information processing Spatial delayed response	Veasey et al. [24, 27] Komiyama et al. [26] Kotopoulea-Nikolaidi et al. [25]
Cognitive flexibility	Shift Stroop	Kotopoulea-Nikolaidi et al. [25]

### Results of individual studies

The synthesised results showed that there was no indication that effects of exercise on cognitive performance, measured either during or shortly after exercise, are altered by prior breakfast consumption or macronutrients of breakfast [25, 26]. Specifically, one study found that 30-minutes of moderate-intensity cycling with and without prior breakfast both induced faster basic information processing assessed during exercise relative to baseline in healthy, active young men [26]. Interestingly, the accuracy of information processing appears to increase in response to fasted exercise [26]; a response that was not seen during Breakfast-Ex condition [26]. Another study showed no difference across fasted high-intensity intermittent uphill walking/jogging and the same exercise with either high-carbohydrate or high-protein breakfast beforehand in exercise induced improvements in inhibitory control and cognitive flexibility in overweight perimenopausal women [25].

There were neither effects of exercise nor breakfast-exercise interaction on sustained attention [27], divided attention [23], and working memory [24–27]. By combining either overnight fasting, corn breakfast, wheat breakfast, or oat breakfast with a prolonged bout (i.e., 90 min) of continuous cycling at moderate intensity, Paul et al. found neither effects of exercise nor breakfast-exercise interaction on information processing and divided attention [23]. Two studies by Veasey and colleagues [24, 27] combined moderate-intensity treadmill running with either high-caloric breakfast, low-caloric breakfast, or overnight fasting and, again, found null effect of exercise or breakfast-exercise interaction on information processing, sustained attention, inhibitory control, and working memory in young healthy adults.

### Methodology quality

The studies that were incorporated showed a ‘*fair*’ methodological quality as denoted by their mean PEDro score of 4.0. All of the studies included in the analysis reported implementing random participant allocation procedures for their respective trials, suggesting low allocation bias. None of the studies mentioned blinding of the participants, therapists and assessors. Each of the studies included in the analysis provided a primary outcome measure for over 85% of their participants, and all studies adopted a crossover trial design. However, none of the studies explicitly reported conducting an intention-to-treat analysis. This analysis involves including at least one primary outcome in the statistical analysis for all participants, irrespective of any withdrawals from the treatment. All the studies compared the outcomes of two or more groups/conditions in a study. Lastly, the data on point and variability measures were not reported in two studies, whereas the remaining three studies did provide this information. The details of the PEDro evaluation are displayed in Table 3.

**Table 3:** Summary of risk of bias assessment

Study	Eligibility	Random allocation	Concealed allocation	Similar baseline	Blinding subjects	Blinding therapists	Blinding assessors	85% retention	Intention-to-treat	Between group comparison	Point and variability measures	Total score
Paul et al. [23]	YES	YES	NO	NR	NO	NO	NO	YES	NO	YES	NR	3
Veasey et al. [27]	YES	YES	NO	NR	NO	NO	NO	YES	NO	YES	NR	3
Veasey et al. [24]	YES	YES	NO	YES	NO	NO	NO	YES	NO	YES	YES	5
Komiyama et al. [26]	YES	YES	NO	NO	NO	NO	NO	YES	NO	YES	YES	4
Kotopoulea -Nikolaidi et al. [25]	YES	YES	NO	YES	NO	NO	NO	YES	NO	YES	YES	5
Mean score (SD)	4.0 (1.0)											

*Note.* NR = not reported; Scoring: YES = 1, NO = 0; the following cut-points were used to describe the quality of papers: 9–10 (excellent), 6–8 (good), 4–5 (fair), ≤ 3 (poor)

Item one, eligibility, was not included to calculate the PEDro score

## Discussion

The purpose of this study was to review whether morning breakfast consumption/ omission before exercise would moderate cognitive performance assessed during or following exercise. In contradiction to our hypothesis, the synthesised results based on 5 identified studies showed that there was no indication that effects of exercise on cognitive performance (e.g., processing speed, inhibitory control), either assessed concurrently with exercise or shortly after exercise, are altered by breakfast skipping and/or consumption (e.g., different portion, macronutrients, and contents) in healthy adults. However, the current synthesised results should be interpreted cautiously due to high heterogeneity of the research protocols and limited studies with mostly young, healthy adults.

### The interaction effects of breakfast consumption and exercise on cognition

Preliminary data assessed in this review suggests that breakfast omission and/or consumption prior to an exercise bout may not modulate the effects of exercise on cognitive performance, either assessed during or shortly after exercise. For instance, while 30 min of moderate-intensity cycling with(out) morning breakfast consumption both induced faster information processing assessed during exercise in healthy, active young men, there was no difference in exercise-induced changes in information processing as a function of breakfast [26]. It is also noteworthy that participants had higher accuracy when responding to the Go trials immediately after breakfast consumption as compared to overnight fasting, suggesting that breakfast consumption transiently improves information processing and vigilance. This finding corroborates findings from previous studies who indicated that breakfast consumption can improve performance on tasks components tapping vigilance and basic information processing, and this effect could be explained by increased blood glucose [20]. The higher response accuracy immediately following breakfast may partly explain the unchanged performance from pre-exercise to during-exercise cognitive assessment, as it may be plausible that there was no capacity for improvement; in contrast, there was improvement in response accuracy of the Go trials during the fasted exercise condition due to worse performance before exercise.

Respective to effects of different macronutrients of breakfast, the included studies indicated no moderating effect of such factor to post-exercise cognitive performance. Specifically, whereas performance on inhibitory control and cognitive flexibility (but not working memory) were improved by acute 30-minute bouts of high-intensity interval exercise in perimenopausal women, the exercise induced improvements were not differed across a fasting-exercise condition, a high-carbohydrate breakfast-exercise condition, and a high-protein breakfast-exercise condition [25]. These findings suggest selective effects of acute high-intensity interval exercise on inhibitory control and cognitive flexibility in women, and these selective effects would not be differed either by pre-exercise breakfast or the macronutrients of breakfast. In terms of performance on higher-order cognition, such as executive function, it remains debatable whether different macronutrients of breakfast would exert differential effects [32, 33], and a few studies further indicated that individuals’ glucose tolerance and the time course of glucose fluctuations after meal could play a key role in affecting post-breakfast cognitive performance [34]. This might explain the null interaction effect between breakfast and exercise in the Kotopoulea-Nikolaidi et al. study.

It should be noted that 3 from the 5 studies found neither an effect of moderate-intensity aerobic exercise (ranging from 30 to 90 min) nor a breakfast-aerobic exercise interaction on information processing, sustained and divided attention, inhibitory control, and working memory assessed following exercise [23, 24, 27]. While the null effects of breakfast consumption are in alignment with the abovementioned 2 studies [25, 26], the non-significant effects of acute bouts of exercise are in contradiction to the extant evidence [35, 36] and could be explained by several factors. First, age has been shown to be a key factor moderating the effects of acute exercise on cognition, with children and mid-age and older adults experiencing greater benefits from exercise to executive function as compared to young adults [35, 36]. It is plausible that participants (i.e., mid-age perimenopausal women) in the Kotopoulea-Nikolaidi et al. study experienced greater exercise exerted benefits to inhibitory control and cognitive flexibility as compared to studies that focused on young healthy adults [23, 24, 27]. Another factor could be the target cognitive domain. According to the Reticular-Activating Hypofrontality (RAH) model, performance on sensorimotor speed, visual attention, and alertness can be improved during exercise to favour exercise performance [37]; once the exercise stopped and a dual-task situation no longer exists, the effects of exercise on lower-order cognitions weakens while the effect on executive function becomes stronger [38, 39]. These modulations may explain why performance on processing speed, visual search, and attention were not altered by acute exercise studies by Paul et al. and Vessey et al. and can be supported by studies who found disproportionally larger acute aftereffects of exercise to executive function relative to basic information processing [36].

As to why Breakfast-Ex yields no stronger effects on cognition than Fasted-Ex, we speculated that the switch between glucose and ketone bodies as energy sources could be one potential explanation. Specifically, while our brain majorly relies on glucose for functioning, it has been found that ketone bodies, a product of lipid metabolism, could be an alternate energy source, especially during a fasting state [40, 41]. Studies showed that exercise under a fasted state demonstrated greater lipid metabolism and elevated ketone bodies (e.g., β-hydroxybutyrate) [42, 43] and increased ketone bodies may, in turn, maintain cognitive functioning during and following exercise despite lack of energy intake (e.g., breakfast) combined with reduced glycogen storage resulted from Fasted-Ex. This assumption is hypothetical, and more research are needed to verify our speculation. The synthesised findings regarding the null effects of morning breakfast consumption and/or macronutrients of breakfast may have practical implications to healthy, non-clinical individuals. For example, for healthy office workers who need to walk or cycling to the office but do not have time for breakfast (or forget to have breakfast) in the morning, omission of breakfast may not acutely compromise their work performance within the first few hours following exercise. Relatedly, for athletes who have training sessions in the morning, it is possible that omission of breakfast (commonly occurred whilst early morning training under a fasted state) would not significantly affect their response speed and vigilance during training.

Apart from the abovementioned explanations, from a statistics point of view, it is also plausible that either the null effects of exercise on cognition or the non-significant interaction between exercise and breakfast on cognition simply reflects a Type 2 error introduced to statistical analyses in the included studies due to small sample size and low statistical power.

### Limitation of published research and future directions

Some caution is warranted when interpreting the findings of this review. First, the results cannot be generalised beyond the specific populations studied, that is, children and adolescents, individuals with metabolic disease or with obesity, or individuals with cognitive impairments. The current literature has rarely focused on youth populations. Notably, one recent study found that breakfast (taken after exercise) and exercise combine improved mathematical task performance in adolescents [44]. More research on youth population should be warranted to develop a healthy lifestyle arrangement that can be adapted in school settings. In addition, individuals with metabolic disease (e.g., obesity) [45] and cognitive impairment (e.g., mild cognitive impairment) [46], as well as those who enter post-menopausal stage [47], show worse cognitive performance than their healthy counterparts. Inferior performance at baseline may give these individuals greater space for improvement as a function of either breakfast or exercise. Indeed, one recent study showed differential cognitive responses to exercise between children with normal weight and their obese peers [48]. It is essential to independently examine the interactive effects between breakfast and exercise on cognition in across these individuals. Second, as noted in methods and results, we were only able to extract cognitive measures that were not preceded by non-breakfast related energy intake, which only affords us to evaluate the effects of breakfast and exercise on cognitive performance up to 2 h following exercise termination. This means that the prolonged effects of breakfast consumption/omission and exercise on cognition remains unclear. Third, it should also be noted that the typical duration for a cognitive task would last no longer than 30 min per task. As such, the effects of breakfast consumption/omission and exercise on cognitive-focused tasks lasting longer than 30 min, such as many job- or school-related tasks in real-world scenarios, remains unclear. Fourth, it is noteworthy that most of the included studies did not have a non-exercise control condition. Without a non-exercise control condition, whether the observed changes in cognition were driven by practice effects or familiarisation remain unknown. Fifth, given the heterogeneity of exercise protocols, contents of breakfast, cognitive assessments, characteristics of participants, as well as small sample size across the included studies, more studies with a statistically robust sample size are needed to systematically clarify the role of exercise protocols (e.g., modality, intensity, duration), contents and macronutrients of breakfast (e.g., high-carbohydrate, high-protein), and participants’ characteristics (e.g., normal weight, obese) on the interaction effects of breakfast and exercise on different domains of cognition. Relatedly, the limited number of available studies and high heterogeneity across studies precluded us from running meta-analysis and synthesising the results quantitatively. However, our narrative synthesis still provides relevant updates on the existing literature and highlights areas for improvement and future prospects. Lastly, there is a need to further explore the underlying mechanisms using biochemical and physiological measures, such as activity of the LC-NE system captured by neuroimaging measures (e.g., EEG, pupillometry), glucose and fat metabolism, serum levels of BDNF.

## Conclusions

The current review provides preliminary evidence that the pre-exercise morning breakfast consumption and/or omission may not moderate the effects of acute exercise on cognition assessed either during exercise or up to 2 h post-exercise. However, the current synthesised results should be interpreted cautiously due to high heterogeneity of the research protocols, limited studies with mostly young and healthy adults included. The findings presented in this study may not be applicable to individuals with chronic metabolic diseases or other medical conditions.

### Practical implications

- For healthy individuals, morning breakfast or overnight fasting does not seem to affect the effects of exercise on performance in vigilance, attention, and executive function, at least within the first 2 h following exercise.
- For healthy office workers who need to walk or cycling to the office but do not have time for breakfast in the morning, the omission of breakfast may not acutely compromise their subsequent work performance (within the initial 2 h).

## Electronic supplementary material

Below is the link to the electronic supplementary material.

Supplementary Material 1

Supplementary Material 2

Supplementary Material 3

## Data Availability

The statement provided in the manuscript.

## Abbreviations

PRISMA: Preferred Reporting Items for Systematic Reviews and Meta-Analysis
PROSPERO: International Prospective Register of Systematic Reviews
PICOS framework: The Population, Intervention, Comparison, Outcome and Study framework
PEDro: Physiotherapy Evidence Database scale
EF: Executive functions
BDNF: Brain-derived neurotrophic factors
TNFα: Tumour necrosis factor-Alpha
IL-6: Interleukin 6
EEG: Electroencephalogram
