# Supplementary material for: Systematic review on the effects of exercise with and without breakfast consumption on cognitive performance in healthy adults
Source: BMC Psychol. 2025 Jan 10;13:29. doi: 10.1186/s40359-024-02327-y (PMC11720559; doi:10.1186/s40359-024-02327-y)
Supplement: Supplementary file 3 — Supplementary Material 3 [file 40359_2024_2327_MOESM3_ESM.docx]

| Concept | Words/synonyms for each concept | Truncation use |
| --- | --- | --- |
| Breakfast | Fast  **OR**  Fasting  **OR**  Carbohydrate | Breakfast  Fast*  Carbohydrate |
| AND | | |
| Exercise | Physical activity | Exercise  Physical activit* |
| AND | | |
| Cognition | Cognitive function  **OR**  Executive function  **OR**  Memory | Cognition  Cognitive function  Executive function  Memory |

**Search plan: keywords, alternatives and truncation**

**Web of Science**

**Techniques used:**

* for truncation

“ ” for phrases (finding words next to one another)

( ) brackets around sets of words for each concept

**Search statement:**

("breakfast" OR "fast*" OR "carbohydrate") AND ("cognition" OR "cognitive function" OR "executive function" OR "memory") AND ("exercise" OR "physical activit*")

Results = 533 results

**Scopus**

* for truncation

Wildcards not supported so need to include more alternatives manually

( ) brackets around sets of words for each concept

**Search statement:**

(breakfast OR fast* OR carbohydrate) AND (cognition OR {cognitive function} OR {executive function} OR memory) AND (exercise OR {physical activity} OR {physical activities})

Additional filters applied: published in English, journal articles, full-text available

Results = 694 results

**PubMed**

**Techniques used:**

* for truncation

“ ” for phrases (finding words next to one another)

( ) brackets around sets of words for each concept

**Search statement:**

((((("breakfast"[Title/Abstract] OR "fast*"[Title/Abstract] OR "carbohydrate"[Title/Abstract]) AND ("cognition"[Title/Abstract] OR "cognitive function"[Title/Abstract] OR "executive function"[Title/Abstract] OR "memory"[Title/Abstract]) AND ("exercise"[Title/Abstract] OR "physical activit*"[Title/Abstract])) NOT "review"[Title/Abstract]) NOT "meta analy*"[Title/Abstract])

Additional filters applied: abstract and full text available

Results = 312 results

**Medline**

**Techniques used:**

* for truncation

‘ ‘ for phrases (finding words next to one another)

**Search statement:**

**#1** ‘breakfast’ OR ‘fast*’ OR ‘carbohydrate’

**#2** ‘cognition’ OR ‘cognitive function’ OR ‘executive function’ OR ‘memory’

**#3** ‘exercise’ OR ‘Physical activit*’

**#4** #1 AND #2 AND 3

Results = 501 results

**Embase**

**Techniques used:**

* for truncation

‘ ‘ for phrases (finding words next to one another)

**Search statement:**

**#1** ‘breakfast’ OR ‘fast*’ OR ‘carbohydrate’

**#2** ‘cognition’ OR ‘cognitive function’ OR ‘executive function’ OR ‘memory’

**#3** ‘exercise’ OR ‘Physical activit*’

**#4** #1 AND #2 AND 3

Additional filters applied: Only on Embase, Participants are humans, Published in English, Articles

Results = 978
